# Supplementary material for: A Salmonella Typhi Controlled Human Infection Study for Assessing Correlation between Bactericidal Antibodies and Protection against Infection Induced by Typhoid Vaccination
Source: Microorganisms. 2021 Jun 28;9(7):1394. doi: 10.3390/microorganisms9071394 (PMC8304662; doi:10.3390/microorganisms9071394)
Supplement: Supplementary file 1 [file microorganisms-09-01394-s001.zip › microorganisms-1244450-supplementary.pptx]

## Slide 1
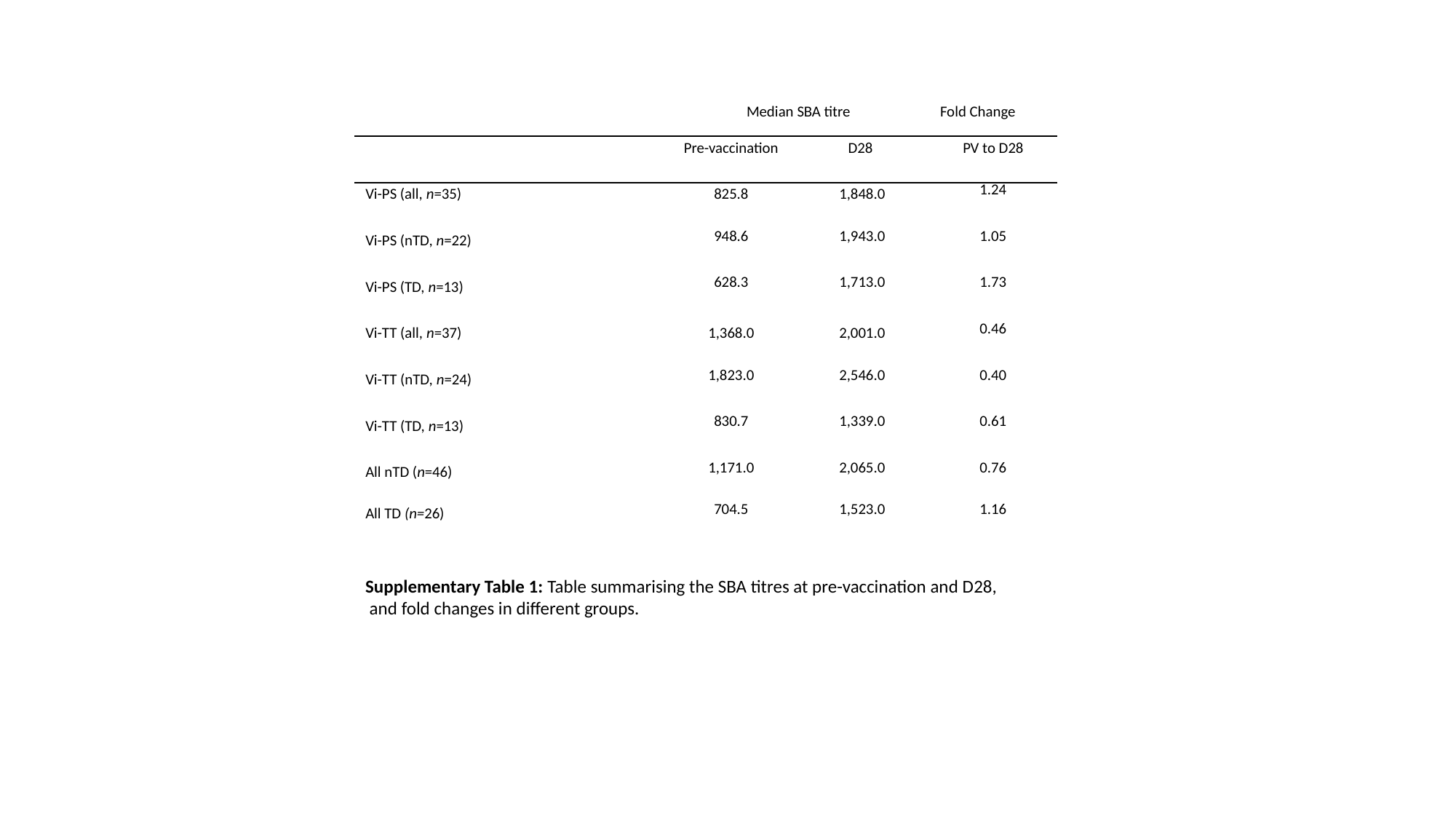

| | Median SBA titre | | Fold Change |
| --- | --- | --- | --- |
| | Pre-vaccination | D28 | PV to D28 |
| Vi-PS (all, n=35) | 825.8 | 1,848.0 | 1.24 |
| Vi-PS (nTD, n=22) | 948.6 | 1,943.0 | 1.05 |
| Vi-PS (TD, n=13) | 628.3 | 1,713.0 | 1.73 |
| Vi-TT (all, n=37) | 1,368.0 | 2,001.0 | 0.46 |
| Vi-TT (nTD, n=24) | 1,823.0 | 2,546.0 | 0.40 |
| Vi-TT (TD, n=13) | 830.7 | 1,339.0 | 0.61 |
| All nTD (n=46) | 1,171.0 | 2,065.0 | 0.76 |
| All TD (n=26) | 704.5 | 1,523.0 | 1.16 |
Supplementary Table 1: Table summarising the SBA titres at pre-vaccination and D28,
 and fold changes in different groups.
